# Food Preferences in Dogs: Effect of Dietary Composition and Intrinsic Variables on Diet Selection

**DOI:** 10.3390/ani9050219

**Published:** 2019-05-06

**Authors:** Raúl A. Alegría-Morán, Sergio A. Guzmán-Pino, Juan Ignacio Egaña, Carem Muñoz, Jaime Figueroa

**Affiliations:** 1 Departamento de Medicina Preventiva Animal, Facultad de Ciencias Veterinarias y Pecuarias, Universidad de Chile, Santiago 8820808, Chile; ralegria@veterinaria.uchile.cl; 2 Departamento de Fomento de la Producción Animal, Facultad de Ciencias Veterinarias y Pecuarias, Universidad de Chile, Santa Rosa 11735, La Pintana, Santiago 8820000, Chile; sguzmanp@uchile.cl (S.A.G-P.); jegana@uchile.cl (J.I.E.); carem.vet@gmail.com (C.M); 3 Departamento de Ciencias Animales, Facultad de Agronomía e Ingeniería Forestal, Pontificia Universidad Católica de Chile, Santiago 7820436, Chile

**Keywords:** domestic dog, food preference, intrinsic variables, nutrients, pet food

## Abstract

**Simple Summary:**

Preference tests allow the study of some aspects of the feeding behavior of domestic dogs. However, besides sensorial proprieties of food, intrinsic characteristics of dogs may play a role in diet selection. The aim of this study was to describe the feeding behavior of dogs in relation to diet composition and their intrinsic variables (age, breed, sex, and body weight) by using a ten-year database of two-feeder food preference tests (2007–2017). The content of less digestible fractions like crude fiber and dry matter negatively affected dogs’ food preferences. In addition, animals’ weight and breed influenced dogs’ intake of the most preferred diets, where heaviest dogs presented lower intakes as did the Beagle breed in relation with both Boxer and Labrador Retrievers. Moreover, the hot season of the year decreased dogs’ intake of preferred diets. In terms of preferences, Boxers presented a lower preference for selected diets compared to the other breeds. Finally, age and sex did not affect dogs’ preference or intake of the foods selected. The understanding of the relationships between food composition and intrinsic variables of dogs with their diet selection could improve specific pet food formulation in order to satisfy animals’ physiological and hedonic needs.

**Abstract:**

A ten-year food preference database (2007–2017) was used to relate food selection in dogs to the nutritional components of diets by doing a principal component analysis (PCA) and a linear regression between components obtained and dogs’ preferences. Intake and preference of preferred diets were analyzed by dogs’ sex, breed, age, body weight, and the season of the year (hot or cold). The fourth component after PCA presented a relation with food preferences (OR = −2.699, *p* = 0.026), showing negative correlations with crude fiber (*rho* = −0.196; *P* = 0.038) and dry matter (*rho* = −0.184; *p* = 0.049). Weight (OR = −1.35; *p* < 0.001), breed, both Boxer (OR = 10.62; *p* = 0.003) and Labrador Retriever (OR = 26.30; *p* < 0.001), and season (hot season) (OR = −5.27; *p* < 0.001) all influenced animals’ intake. Boxers presented a lower food preference compared to the other breeds (OR = −44.3; *p* < 0.001), while animals’ weight influenced preferences only in Boxers (OR = 2.02; *p* < 0.001). Finally, age and sex did not affect dogs’ preference or intake of preferred diets. Thus dry matter and fiber content have a negative impact on dogs’ food choices. Dogs’ weight, breed, and season affected food intake, but only breed affected dogs’ preferences, which is probably explained by adaptive changes in the detection, metabolization, and learning of nutritive food cues.

## 1. Introduction

Food preference tests are used as an effective tool to estimate some aspects of the feeding behavior in dogs [1,2], contributing to new diet formulations to meet animals’ nutritional and palatability requirements. In preference tests, dogs are simultaneously exposed to different diets over a certain amount of time (which is usually shorter in dogs than in other species, because of their voracity) [3]. Dietary preferences in mammals are intimately linked to the sensorial characteristics of foods, reflecting, in part, their nutrient composition. In this light, it has been described that dogs are able to select and prefer foods even when they liberate small amounts of volatile compounds, enhancing consumption when there is more moisture content and a small particle size [4,5]. Similarly, although the dog maintains strong preferences for protein foods [6], domestication has created adaptive physiological changes that have generated preferences for simple carbohydrates [7], such as sucrose [4,8]. In addition, dietary preferences in dogs are influenced not only by the sensorial or nutritional proprieties of foods, but also by intrinsic variables of animals [9]. Alliesthesia could affect the intake or preference of dogs for specific nutrients or related flavors depending on their internal status that generates different pleasure sensations [10]. Thus, variables such as a dog’s breed, age, body condition, weight, or sex may affect their feeding behavior. However, there is little information about how all these variables may affect food choices in domestic dogs. Knowing the effect of these variables allows a better understanding of their food selection and food acceptability. The aim of this study was to understand the relationship between the nutritional compositions of dogs’ diets and their associated preferences, and to analyze how intrinsic variables of these animals may influence their feeding behavior.

## 2. Materials and Methods

A database of ten years (2007–2017) of dogs’ food preference tests was used to analyze the effect of diet composition and the intrinsic variables of dogs on their food intake and preferences. Data was obtained from the Research Center of Pet Feeding Behavior of Facultad de Ciencias Veterinarias y Pecuarias (FAVET), Universidad de Chile, located in the Metropolitan region of Chile (Santiago city, 34°21′ S, 71°18′ W). Experimental procedures were performed according to the specifications of the bioethical committee of FAVET (N°042013 as an example).

### 2.1. Animals and Housing

A total of 1771 preference tests using 34 different kennel dogs (27 male and 7 female) were used in this study over the study period (2007–2017). An average of 16 male and female unneutered dogs (*canis familiaris*) were maintained per year at the Center during the studied period. Animals corresponded mainly to three different breeds (Beagle, Labrador, and Boxer), predominantly Beagles (70.5%). Animals were obtained at an average of six months of age from different commercial breeders, making sure they were not genetically related, and they were subjected to a clinical evaluation by veterinarians of the Clinical Sciences Department of FAVET before being registered. A training period of six months was applied to all new animals, helping them to get used to the experimental procedures. During this period, they were adapted to two-feeder preference trials with highly palatable foods in both feeders to stimulate the intake of the two options. Afterwards, they performed the same experimental trials as adult dogs but their data was not used for the experimental purposes until the end of the training period. Health of dogs was assessed prior to the start of the experiments through clinical, hematologic, and biochemical serum profiles, and all dogs that participated in preference trials were considered healthy. The age of dogs performing preference tests ranged from 1 to 15 years old, where males usually represented a greater percentage of total animals. Dogs were housed individually in single kennels with internal (1.65 × 1.85 m) and external (1.65 × 2.45 m) rooms. In the internal room, dogs were fed daily at 09:30 h a commercial extruded food presented in a single ration (3% of their body weight). The Center provided partial control of environmental conditions by door and window management. However, average temperature varied across different seasons during the year, ranging (approximately) from 22.0 °C during hot season to 7.7 °C during cold season [11]. Animals had ad libitum access to fresh water and a comfortable bed. Dogs were exercised daily for approximately 60 min during the afternoon in an open field of the campus by veterinary students. Moreover, animals had access to an external field where they could join other dogs from the kennel each day during the morning and afternoon for approximately 45 min. Dogs regularly received clinical examinations by trained personnel from FAVET. Dogs’ body weight was monitored monthly in order to adjust the amount of food offered and maintain a stable body condition. In general, dogs ended their participation in experimental trials when they reached 7 years of age (with some exceptions) or when they presented diseases not compatible with the preference tests or that could alter their results. 

### 2.2. Preference Procedures and Database

Trials were performed in the kennel and consisted of food preference tests that evaluated variations in the nutritional, physical, and/or organoleptic characteristics of foods. Preference tests were performed in the internal room during the morning (09:00 h). Tests consisted of the simultaneous placement of two feeders in the front of the dog’s kennel for 20 consecutive minutes and usually lasted 4 days (1 test/day) in which diets were counterbalanced in position (left or right) to diminish the effect of possible side bias over preferences. As well as the daily administration of food during nontrial periods, foods offered during the tests were adjusted to 3% of the dog’s body weight in each feeder. On experimental days, animals were fed only the experimental diets at the moment of the test. If an animal consumed the entire amount offered in one feeder during the preference test, both feeders were immediately removed, and the consumption was evaluated. To estimate the amount consumed, feeders were weighed at the beginning and the end of each test. In order to equilibrate intake of animals and diminish size effects, consumption was subsequently corrected by their metabolic weight (total consumption/live weight^0.75^). Nutritional composition of both diets was analyzed by proximal chemical analysis, acid hydrolysis, and calorimetric pump (IKA^®^ calorimetric system c 2000, USA). Diets were analyzed following standard Association of Official Analytical Chemist (AOAC) methods [12] for content of moisture (method 945.15), crude protein (CP, Kjeldahl method 945.18, N x 6.25), ether extract (EE, method 945.16), ash (method 920.153), crude fiber (CF, method 962.09), total lipids (LIP, method 922.06), calcium (Ca), phosphorus (P), dry matter (DM), nitrogen-free extract (NFE), and metabolizable energy (ME).

With the objective to homogenize and compare different studies performed across the whole period (2007–2017) without having the same standard diet across tests, diets were defined as Diet A (most preferred) and Diet B (less preferred) in each test, and preferences were estimated by the relative consumption of Diet A over Diet B ((intake of Diet A/{intake of Diet A + intake of Diet B}) x 100) [13], allowing evaluation of the effect of variables on the selection of the most preferred diet (Diet A). Consumption and preferences for Diet A were ordered by dogs’ sex, breed, age, weight, and season at the moment of each test for statistical analysis.

### 2.3. Statistical Analysis

#### 2.3.1. Effect of Nutrient Composition Over Dogs’ Food Preferences

To evaluate how nutritional components of diets (DM, CP, CF, EE, NFE, ash, Ca, P, LIP, and ME) may explain food preferences and if the components were grouped, a principal component analysis (PCA) was carried out using ”devtools” and “ggbiplot” packages of the statistical software R [14]. Nutritional components were expressed as the net difference between Diet A and B, with the exception of metabolic energy, which was expressed as the percentage difference between diets. After the PCA, a multiple linear regression was performed between the most important variables grouped in the PCA and dogs’ preferences by using R statistic software. Later, Spearman correlations were performed with the nutritional components that represented the greatest variability within the component that showed a significant effect at the multiple linear regression.

#### 2.3.2. Effect of Intrinsic Variables Over Dogs’ Food Preferences

The effect of intrinsic variables on intake and preference of Diet A was investigated using linear mixed models. These models, also known as variance component models, take into account the nested structure of a dataset and provide the chance to decompose the variance into a number of components which can then be given a proper and useful interpretation [157].

The intrinsic variables recorded in this study correspond to three categorical variables, sex (male or female), breed (Beagle, Boxer, or Labrador), and age (<5 years or >5 years), with body weight registered as a continuous variable. Season was also recorded, considering cold season as all the experiments performed between 21 March and 20 September (autumn and winter seasons in the southern hemisphere) and hot season all the experiments performed between 21 September and 20 March (spring and summer seasons in the southern hemisphere).

Two models were developed in order to study the effect of the registered variables over intake and preference of Diet A in dogs. Dog variable was treated as a random effect, while the rest of the variables were treated as fixed effects. The proposed model to explain intake and preference is:

(1) $$Y_{km}=\beta_{0}+\beta_{n}+b_{k}+\varepsilon_{m\left(k\right)}.$$

where *Y_km_* correspond to the corrected intake or to the preference of Diet A, *β*_0_ is the intercept, *β*_n_ is the vector of fixed effects, *b*_k_ is the random effect of the kth dog, and ε_m(k)_ is the error term. The model assumes that random effects and errors are independent at the nesting level and follow an approximately normal distribution, with a mean equal to zero and variance equal to σ^2^.

Fixed effects were selected from the initial model (full model) using Likelihood Ratio Test (LRT) and removing those variables whose regression coefficients were not significant (*p* > 0.05) using a backward elimination procedure [18]. Nonsignificant variables that when removed produced a change of ≥20% in the regression coefficients of the significant variables were retained in the model in order to adjust for confounding factors [17]. Fixed effects corresponding to categorical variables (e.g. Breed) were entered to the model as dummy variables, leaving one level as reference. With this, the regression coefficients from the levels of categorical variables must be interpreted with respect to their reference level. In order to estimate possible differences between levels of a variable that were not directly compared with each other, an adjusted mean comparison test was done, using the Di Rienzo, Guzmán, and Casanoves test with a significance level *p* < 0.05 [19]. All of the analysis was carried out using “lmerTest”, “mgcv”, “e1071” packages of the statistical software R [14].

## 3. Results

### 3.1. Effect of Nutrient Composition on Dogs’ Food Preferences

**Figure 1:**
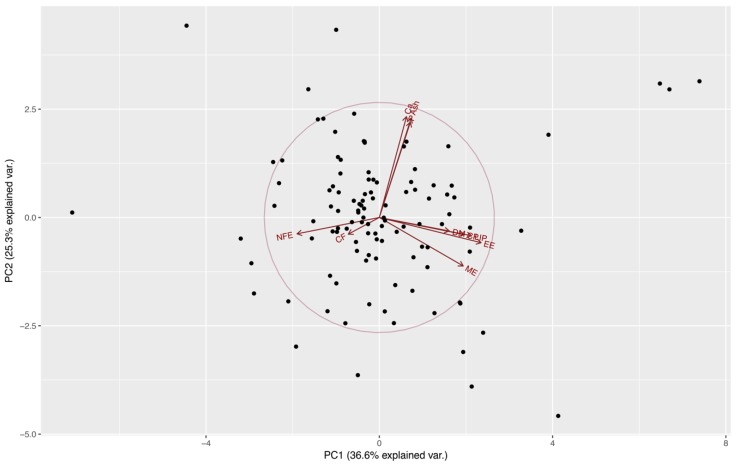
Distribution of food preference of kennel dogs, on the first two principal components extracted from estimate contents of dry matter (DM), crude protein (CP), crude fiber (CF), ether extract (EE), nitrogen-free extract (NFE), ash, calcium (Ca), phosphorus (P), total lipids (LIP), and metabolizable energy (ME) by proximal chemical analysis, acid hydrolysis, and calorimetric pump food decomposition.

From Table 1 we can observe that the first four components all have variances (eigenvalues) greater than 1 and together account for almost 85% of the variance of the original variables (36.61, 25.26, 12.53, and 10.31%, respectively). These components might be used to summarize the data in the multiple linear regression analyses with little loss of information. Eigenvectors (Table 1) from these components confirm the presence of a correlation among some of the nutrients evaluated, also observed at an individual correlation test (results not published). This indicates an aggregation of components in this case in four major groups: lipid component (LIP and EE) at PC1, a mineral component (Ca, P, and ash) at PC2 (Figure 1), and the importance of DM and CF at PC3 and PC4, respectively.

After the multiple linear regression between these four principal components and dogs’ food preference (Diet A), a significant relationship was observed only with the fourth principal component (β = −2.699, *p* = 0.026) (Table 2). This principal component is dominated by the effect of CF (Table 1), indicating that higher levels of this element in the diet affect negatively the preference of dogs. The Spearman’s correlation test showed negative statistically significant correlations between preference and CF, and DM (rho = −0.196, *p* = 0.038, and rho = −0.184, *p* = 0.049, respectively).

### 3.2. Effect of Sex, Breed, Age, Body Weight, and Season on Dogs’ Food Intake

Table 3 shows fixed effects retained in the final intake model and their parameter estimators. Those that exhibited a significant association with dog food intake were: weight, breed, and season. Sex was retained in the final model despite not showing a significant association with dog food intake, because it appeared to be a confounder with weight.

In the case of weight, it can be seen that there was a significant association with food intake (*p* < 0.001), indicating that for each unit of increase in weight, food intake decreased by 1.35 units of intake g/animals weight^0.75^. In the case of categorical variables, again breed, the reference level remained the same (Beagle). It can be seen that Boxers and Labrador Retrievers presented a significant association with food intake (*p* = 0.003 and *p* < 0.001, respectively), indicating that Boxers and Labrador Retrievers had an increase in intake of 10.62 and 26.30 units of intake g/animals weight^0.75^ respectively, compared with Beagles. When comparing the adjusted means of the three breeds, the effect on food intake was consistent, showing significant differences between them (Table 4). Finally, in the case of season, the reference level corresponded to the cold season. It can be seen that the hot season presented a significant association with food intake (*p* < 0.001), indicating that food intake decreased by 5.27 units of intake g/animals weight^0.75^ compared with the cold season. 

### 3.3. Effect of Sex, Breed, Age, Body Weight, and Season on Dogs’ Food Preferences

Table 5 shows fixed effects retained in the final food preference model and their parameter estimators. Those that exhibited a significant association with dog food preference were breed and the interaction between weight and breed. Weight, sex, and season were retained in the final model despite not showing a significant association with dog food preference, because they appeared to be confounders with the significant variables.

In the case of categorical variables, taking the case of breed, the regression coefficients represent the effect of a particular level of the variable on food preference, compared to a level chosen as the reference (Beagles, in this case), given the other variables are held constant. It can be seen that Boxers presented a significant association with food preference (*p* < 0.001), presenting a food preference of 44.3 percentage units less than Beagles. When comparing the adjusted means of the three breeds, the effect over food preference is not consistent, showing no significant difference between them (Table 6). For the interaction between weight and breed, it can be seen that the interaction with the Boxer breed presented a significant association with food preference (*p* < 0.001), indicating that the heaviest Boxer presented food preferences 2.02 percentage unit higher than the heaviest Beagle.

## 4. Discussion

The present study provides information about possible relationships between food compositions and dogs’ intrinsic characteristics on their food preferences. It is already known that diet selection could reflect the internal needs of an animal, helping them to reach homeostasis [10]. Describing different factors that may influence feeding behavior of dogs can improve diet-specific formulations to increase their palatability and to amend animal health and welfare problems. It was observed from PCA that DM and CF negatively affected dogs’ food preferences, being parts of the third and fourth principal components, respectively. As noted in this study, dogs, as with other mammals, have been reported to prefer moist and semimoist diets to dry diets [4,5]. Nevertheless, de Brito et al. [20] observed that increasing dietary moisture did not affect food preferences of dogs. Nonetheless, it is possible that the difference in moisture level used in that study was not enough to influence preferences. The pet food industry has considerably reduced the water content of their products with dry extruded foods, facilitating their storage and reducing purchase frequency and feed contamination [21]. However, as we observed that DM is negatively related with dogs’ preferences, it is necessary for the industry to include palatable additives to their formulations such as highly digestible ingredients, natural or artificial umami flavors, and/or an external palatable fat cover, which in some cases may disrupt dogs’ intake regulation, leading to obesity problems [22].

It is well known in other mammalian species such as pigs that food digestibility and palatability are closely related, and CF presented a negative relationship with dogs’ preferences [23,24]. The feeding behavior of domestic dogs (*Canis familiaris*) could be described by the knowledge of the feeding patterns of their ancestral species, the wolves (*Canis lupus)* [8], which consist predominantly of the consumption of fresh meat obtained by group hunting with low contents of both DM and CF [6,25]. However, we must also consider that dogs can accept and even prefer different kinds of foods, moving away from their ancestors in the preference for protein-rich foods [26,27]. Conversely, the absence of relationship between PC1, mainly represented by palatable components (CP, EE, LIP, ME) and dogs’ preferences is intriguing. These nutritional components should be key elements when increasing the preference for one food due to their biological value in dogs. Nevertheless, we have to consider that in this study, the type and quality of the protein or fat sources that may change an animal’s preferences were not analyzed [28].

The effect of intrinsic variables such as weight, breed, age, and sex on feeding behavior has been previously studied in several mammals. For example, it is described that humans differ in their dietary preferences explained by some of these factors [29,30]. However, little information was available in the case of the domestic dog [6,31]. In our analysis, breed of dog influenced intake, in which heaviest breeds such as Boxers and Labradors showed higher consumption, even when intakes were corrected by the animals’ body weight. Nevertheless, Beagles were more selective than Boxers as reflected in their higher food preferences across trials. Dog breeds have been developed to perform different activities around human populations, acquiring different motor and cognitive capacities [32,33]. It has been observed that brachycephalic breeds, such as the Boxer, have a lower olfactory capacity due to the morphological changes of their skull that directly affect the position of the olfactory lobe [34], affecting the identification and selection of food. However, it has been observed in a recent experiment that brachycephalic breeds such as Pugs outperformed the German Shepherds in odor discrimination and the Greyhounds in motivation to perform feedings tasks [35]. Bloodhound breeds such as Beagles are commonly used in food preference tests because of their olfactory abilities and for the ease of working with them as small and docile animals [36]. The existence of breeds in different environments may also affect food selection because its availability and cost of acquisition may vary. Even animals genetically related that live in similar environments may develop different feeding behaviors which would be given by a component of "tradition" within the subpopulations [37]. Flavor preferences have been identified due to pre-and postnatal learning in dogs and other species, so this must also be considered to understand their selection of a particular diet [38,39].

Results also showed a relationship between breed and body weight in terms of the animals’ food preferences, with the heaviest Boxers presenting higher preferences than did the heaviest Beagles. Animals’ experience as well as aging could be differently developed in dog breeds. The large amounts of food that dogs are able to consume in a short time [6,25] may predispose some breeds to obesity if they are given diets with high energy or fat content, as in the case of Labradors and Beagles [40]. It is clear that body condition is a better measure than body weight to understand how physiological changes produced by overweight could change food perception and food metabolization, especially when animals have different ages and/or sizes. Nevertheless, the data collection of body condition scores were inconsistent through the years of the study in this experimental center, having used dogs’ weight data that could make more sense in intrabreed comparisons. Obesity may deregulate food intake as well as dietary preferences, and obese animals could need more palatable ingredients to activate neural pathways of pleasure leading to a deterioration in the perception of ingredients that differ little in their reward value. In humans of different body mass indexes (BMIs), there are differences in receptors related to the intensity of lipid perception, affecting preferences for fatty foods [30]. Leptin levels are directly related to body mass, affecting preferences for sweet flavors [41]. A chronic increase in leptin levels has been shown to reduce sweet taste sensitivity, increasing the consumption of sucrose or other sweet components of diets [42]. On the other hand, Boxer breeds tend to maintain their body weight throughout adult life, and lower body weights are probably related to young, less experienced animals. Moreover, because body weight is related to age, especially in the case of Beagles, heavier animals may also suffer changes in preferences due to body changes that could directly affect food selection. A deterioration of dental structure, a reduction of sensorial capacities, a decrease in the energy demand, and other metabolic changes could directly affect the discrimination and selection of food in domestic dogs.

Sex would also have a potential effect on dietary behavior of mammals due to differences in energy needs, olfactory abilities, and hormonal changes among individuals [29]. In dogs, it has been observed that females present a higher preference for diets containing 1% of sucrose and other additives as compared to males [31,43]. The absence of a sex effect over preferences in this study could be explained because previous studies in dogs compared males against sterilized females [31]. The latter tend to accept a wider range of foods, being less selective than males as a result of the suppression of estrogen effects that can increase food intake and appetite [44], suggesting that more than sex influences the effect of animals’ reproductive status over feeding behavior. Finally, similar to behavior observed in several species, dogs were affected by environmental temperature and presented a lower food intake during the hot season of the year [45,46,47,48]. This reflects the animal’s lower energy needs to maintain their temperature in warm environments and should be considered when designing food preference trials to eliminate possible biases, especially when environmental temperature cannot be totally controlled as in the present study. In regards to this, the current results could differ in some way from those performed in completely controlled environments, and may not be able to be extrapolated to all other circumstance. In addition, the care given to the dogs may also differ between experiments, affecting animals’ food preferences. For example, different levels of stress in dogs could change their pleasure perception in front of the diets delivered and thus their preference and acceptability as it was described in other species [49,50,51].

## 5. Conclusions

These results suggest that humidity and fiber content have positive and negative impact, respectively, on dogs’ food preferences as has been noted before in domestic and wild canines. As suggested for other animals, intrinsic variables of dogs such as their weight and breed could change their feeding behavior for palatable foods, something probably explained by differences in the detection, metabolism, or learning of nutritive food cues. These variables need to be considered when designing feeding behaviour trials to improve the nutritional and hedonic quality of commercial diets in dogs according to the physiological characteristics of animals.

## Figures and Tables

**Table 1:** Summary table of principal component analysis (PCA), indicating importance of each nutritional component in the diets preferred in kennel dogs, standard deviation, and the percentage of explanation of variation linked to each principal component.

	Principal Component Eigenvectors									
Nutritional Components^1^	PC1	PC2	PC3	PC4	PC5	PC6	PC7	PC8	PC9	PC10
DM	0.32	−0.07	0.56	0.29	−0.40	0.03	0.19	−0.15	−0.39	−0.35
CP	0.39	−0.09	−0.34	−0.18	−0.58	0.01	0.39	0.14	0.06	0.43
CF	−0.14	−0.09	−0.20	0.90	0.03	0.10	0.17	0.22	0.13	0.13
EE	0.46	−0.14	−0.11	0.13	0.27	0.05	−0.47	−0.05	−0.52	0.41
NFE	−0.37	−0.09	0.59	−0.08	0.08	0.06	0.21	0.03	−0.08	0.66
ASH	0.14	0.54	0.08	0.22	−0.12	−0.29	−0.13	−0.59	0.32	0.26
Ca	0.12	0.55	0.10	0.00	0.13	−0.47	0.11	0.61	−0.21	−0.01
P	0.14	0.52	0.08	−0.04	0.04	0.82	−0.01	0.14	0.06	0.01
LIP	0.42	−0.10	0.03	−0.04	0.62	−0.01	0.60	−0.19	0.15	−0.05
ME	0.38	−0.27	0.39	0.01	−0.01	−0.04	−0.36	0.35	0.62	0.01
	**Principal Component Eigenvalues**									
SD	1.91	1.59	1.12	1.02	0.79	0.58	0.46	0.44	0.36	0.18
% of Variance	36.61	25.26	12.53	10.31	6.30	3.36	2.08	1.90	1.33	0.32
Cumulative %	36.61	61.87	74.40	84.70	91.01	94.36	96.44	98.35	99.68	100.0

^1^ Principal component (PC), dry matter (DM), crude protein (CP), crude fiber (CF), ether extract (EE), nitrogen-free extract (NFE), ash, calcium (Ca), phosphorus (P), total lipids (LIP), metabolizable energy (ME), standard deviation (SD).

**Table 2:** Multiple linear regression with selected components from PCA in relation with food preference of kennel dogs.

Variable	OR^1^	SE^2^	*p*-value
(Intercept)	69.7055	1.2082	<2e−16 ^**^
PC1	0.1554	0.6343	0.8070
PC2	0.8522	0.7636	0.2669
PC3	−1.7891	1.0843	0.1019
PC4	−2.6996	1.1952	0.0259 ^*^

Significance codes: ‘**’ *p* < 0.001; ‘*’ *p* < 0.05; ^1^ Odds ratio or estimation of the impact of the component over food preference; ^2^ Standard error.

**Table 3:** Odds ratio and associated statistic of fixed effects included in the final intake model of kennel dogs.

Variable	OR^1^	SE^2^	*p*-value
(Intercept)	59.63	3.51	<0.001^**^
Weight	−1.35	0.15	<0.001^**^
Sex			
Male	2.62	3.27	0.427
Breed			
Boxer	10.62	3.47	0.003
Labrador Retriever	26.30	5.16	<0.001^**^
Season			
Hot season	−5.27	0.72	<0.001^**^

Significance codes: ‘**’ *p* < 0.001; ^1^ Odds ratio or estimation of the impact of each variable over food intake; ^2^ Standard error.

**Table 4:** Adjusted mean comparison between different kennel dog breeds and seasons for food intake previously corrected by their body weight (intake g/animals weight^0.75^).

Variable	Mean	SE^1^
Breed		
Beagle	36.06^a^	0.84
Boxer	45.92^b^	1.12
Labrador Retriever	50.97^c^	1.90
Season		
Hot	41.34^a^	0.88
Cold	46.65^b^	0.76

Means followed by different superscript in a column indicate significant differences at *p* < 0.05; ^1^ Standard error.

**Table 5:** Odds ratio and associated statistics of fixed effects included in the final preference model.

Variable	OR^1^	SE^2^	*p*-value
(Intercept)	77.02	3.5	<0.001^**^
Weight	−0.09	0.21	0.683
Sex			
Male	−0.99	2.33	0.674
Breed			
Boxer	−44.3	12.77	<0.001^**^
Labrador Retriever	13.72	11.74	0.246
Season			
Hot Season	−1.73	1.26	0.168
Weight:Breed			
Boxer	2.02	0.56	<0.001^**^
Labrador Retriever	−0.29	0.45	0.518

Significance codes: ‘**’ *p* < 0.001; ^1^ Odds ratio or estimation of the impact of each variable over food preference; ^2^ Standard error.

**Table 6:** Adjusted mean comparison between different breeds for food preference (%) of kennel dogs.

Variable	Mean	SE^1^
Breed		
Beagle	75.69^a^	1.22
Boxer	74.51^a^	1.63
Labrador Retriever	76.81^a^	2.77

Means followed by different superscript in a column indicate significant differences at *p* < 0.05; ^1^ Standard error.
